# The Impact of High-Speed Crushing Process of Fibrous Polytetrafluoroethylene on Pyrolyzed Carbon Black/Natural Rubber Composites

**DOI:** 10.3390/polym17020222

**Published:** 2025-01-17

**Authors:** Zheng Gong, Yao Xiao, Yukun Zhou, Donglin Zhu, Baochang Dai, Ziyang Wang, Chuansheng Wang, Huiguang Bian

**Affiliations:** 1 School of Mechatronics Engineering, Qingdao University of Science and Technology, Qingdao 266061, China; zhenggong130@163.com (Z.G.); xiaoyao1562100@163.com (Y.X.); zyk990615@163.com (Y.Z.); zhudonglin9361@163.com (D.Z.); dbcjingyu@163.com (B.D.); ww744662708@163.com (Z.W.);; 2 National Engineering Laboratory of Advanced Tire Equipment and Key Materials, Qingdao University of Science and Technology, Qingdao 266061, China

**Keywords:** natural rubber, composites, polytetrafluoroethylene, pyrolyzed carbon black, wear resistance

## Abstract

This study employed a high-speed rotating crushing process to modify pyrolyzed carbon black (CBp) using self-lubricating and low-friction polytetrafluoroethylene (PTFE). The effects of PTFE content on the dispersion, mechanical properties, wear resistance, and thermal stability of modified PTFE-CBp/natural rubber (NR) composites were investigated. The rotating crushing process from the high-speed grinder altered the physical structure of PTFE, forming tiny fibrous structures that interspersed among the CBp particles. This arrangement encouraged the alignment of CBp particles in specific directions and improved the surface activity of CBp, enhancing the dispersion of CBp within the NR matrix and consequently improving wear resistance. The experimental results indicated that as the amount of PTFE fibers increased, the hardness, wear resistance, and thermal stability of the PTFE-CBp/NR composite significantly improved. Compared to untreated CBp/NR composites, the hardness, modulus at 300%, and wear resistance of the 3 phr PTFE-CBp/NR composites increased by 20%, 24%, 21%, respectively, achieving the preparation of highly wear-resistant CBp/NR composites.

## 1. Introduction

The pyrolysis recycling of used rubber tires plays a significant role in resource reutilization. After pyrolysis, approximately 30% of the products are CBp, with ash content accounting for 20%, and the main component being SiO_2_ [1,2,3]. This CBp is extracted from the rubber of used tires through various pyrolysis methods, such as thermal pyrolysis or vapor-phase pyrolysis [4,5,6,7]. After further processing, CBp can be re-added as an important reinforcing agent in the rubber mixing process. This not only facilitates the secondary utilization of used tire rubber resources but also promotes the recycling of carbon resources, contributing to energy conservation and environmental protection [8].

Polytetrafluoroethylene (PTFE) is a high-performance polymer synthesized from the polymerization of tetrafluoroethylene monomers. It exhibits low friction and excellent self-lubricating properties, significantly improving the friction and wear performance of the matrix materials [9,10]. PTFE also possesses high-temperature resistance, chemical corrosion resistance, and insulating properties, making it widely used in fields such as chemical engineering, aerospace, and machinery [11,12,13]. Park et al. [14] successfully prepared MQ/PTFE composites by mixing PTFE powder with silicone rubber (MQ) using repeated rolling and hot-pressing processes. Mechanical performance tests indicated that when the mass fraction of PTFE was 5%, the tear strength significantly improved, and the tensile properties decreased. Khan et al. [15] filled different grades of PTFE micropowder (MP1100 and MP1200) into ethylene-propylene-diene rubber (EPDM) and conducted tests on physical properties, dynamic mechanics, and friction-wear performance. The mean particle size of MP1100 was 4 µm, with a specific surface area of 7–10 g/m². In comparison, the mean particle size of MP1200 was 3 µm, and its specific surface area ranged from 1.5 to 3 g/m². The results showed that PTFE micropowder effectively enhanced the physical and frictional properties of the material, particularly demonstrating excellent wear resistance. Among them, the MP1200 micropowder reduced the specific wear rate, while the MP1100 micropowder had a more significant effect on improving wear resistance and physical properties. Bai et al. [16] studied the impact of different amounts of PTFE micropowders on the properties of nitrile butadiene rubber (NBR). The experimental results indicated that as the content of PTFE micropowder increased, the mechanical strength of NBR initially rose and then fell, while its cohesiveness gradually decreased. When the PTFE content reached 6 phr, the friction coefficient reached its lowest value, with dynamic and static friction coefficients of 0.33 and 0.57, respectively.

The high-speed grinder is an electrical device that uses high-speed rotating blades to powerfully crush materials. Through the high shear force of the grinder, PTFE and CBp can be thoroughly mixed and interact with each other. The rapidly rotating blades cause the CBp to collide and rub within the chamber, disrupting its original inert structure, causing the lattice to change shape and create small breaks. This causes the ash on the surface of the CBp to fall off, increasing the surface area and exposing more active sites, thus facilitating the physical bonding between CBp and PTFE. Meanwhile, under the influence of high shear forces, the microstructure of the PTFE particles changes, with the polymer chains gradually slipping and stretching to form small fibrous structures [17,18].

Based on this, this research uses a high-speed grinder to blend and modify PTFE and CBp, allowing the low-friction, self-lubricating PTFE to form fibrous structures interspersed among the CBp. The treated CBp with PTFE fibers was characterized using scanning electron microscopy and infrared spectroscopy. Additionally, this study examined the filler dispersion, Payne effect, vulcanization characteristics, mechanical properties, wear resistance, and thermal stability of modified CBp/NR composites with PTFE contents of 1, 3, and 5 phr.

## 2. Materials and Methods

### 2.1. Experimental Materials

Natural rubber (NR) was produced by Hainan Natural Rubber Industry Group Co., Ltd. (Haikou, China). CBp was developed by Cabot Corporation (Boston, MA, USA). Polytetrafluoroethylene (PTFE) was produced by Lue Da Materials Co., Ltd. (Zhengzhou, China). Zinc oxide (ZnO), stearic acid (SAD), accelerator DM, and sulfur were all commercially available industrial-grade products. The materials and their proportions are shown in Table 1.

### 2.2. Experimental Equipment

The model and manufacturer of the equipment used in the experiments are shown in Table 2.

### 2.3. Experimental Program

In this experiment, a high-speed grinder was used for the pre-modification treatment of PTFE-CBp, with PTFE contents of 0, 1, 3, and 5 phr. The corresponding amounts of PTFE were mixed with CBp and crushed for 20 min at a rotational speed of 25,000 rpm. The CBp with 0 phr of PTFE was named “untreated CBp”; the CBp with 1 phr of PTFE was named “PTFE1-CBp”; the CBp with 3 phr of PTFE was named “PTFE3-CBp”; and the CBp with 5 phr of PTFE was named “PTFE5-CBp.” This procedure yielded four different types of CBp, and all mixing processes were carried out using dry mixing methods, ultimately resulting in four groups of CBp/NR composites, labeled A, B, C, and D. A brief explanation of the process is provided in Table 3.

### 2.4. Sample Preparation

#### 2.4.1. Preparation of PTFE-Modified CBp

**Figure 1:**
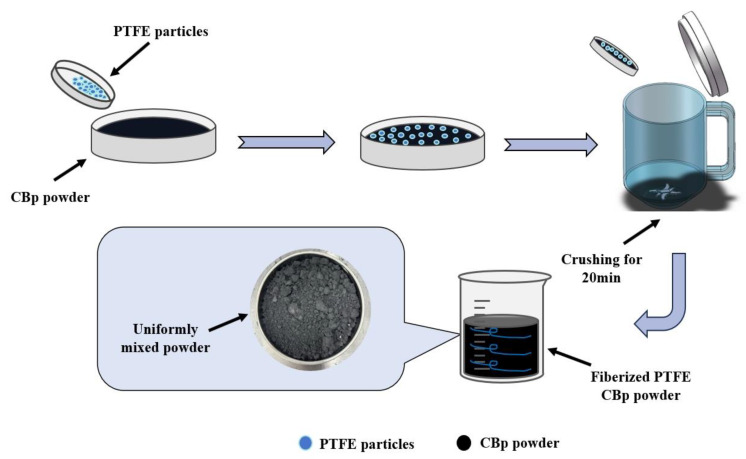
The process flow for the pre-treatment of PTFE-modified CBp using a high-speed grinder.

The pre-treatment process for PTFE-modified CBp using a high-speed grinder is illustrated in Figure 1.

#### 2.4.2. Mixing and Vulcanization

The temperature of the three zones of the internal mixer was set at 100 °C, with a rotation speed of 90 rpm. NR was placed in the internal mixer, and the bolts were dropped. After 1 min and 20 s, CBp, zinc oxide, and stearic acid were added. The bolts were dropped. The bolus was lifted at every minute to clear the leakage of rubber. The total mixing time in the internal mixer was 6 min, and the discharge temperature was 130 °C. Thereafter, accelerator DM and sulfur were added to the open mill. After 12 h of resting, the resulting mixed rubber was placed on the plate vulcanization machine. The vulcanization process was conducted at 150 °C, 11 MPa, and the vulcanization time was set to 1.3 times t_90_ to ensure that the vulcanized rubber fully reached its optimal performance level.

### 2.5. Characterization

#### 2.5.1. Characterization of Properties

The Payne effect was evaluated using a rubber-processing analyzer, with the strain range from 0.28% to 40%, a frequency of 0.01 Hz and a temperature of 60 °C; vulcanization characteristics were measured using a rotorless rheometer, with a test duration of 60 min at a temperature of 150 °C, following the ISO 6502-3:2018 standard [19]; mechanical properties were evaluated using a universal testing machine, following the ISO 37:2005 standard [20], with five samples tested in each group, and the average value was taken; the Mooney viscosity was measured by a Mooney viscometer (PREMIER MV, Alpha Technologies, USA) in accordance with ISO 289-1:2014 [21], with a preheating period of 1 min, a testing period of 4 min, and a testing temperature of 100 °C. The rotor was a large rotor with a diameter of 38 mm; hardness was measured by a H17A Shore A hardness tester, with five values tested for each sample, and the average value was taken as the hardness value; wear resistance was calculated by the mass loss and density of the vulcanized rubber before and after wearing on a rotary roller abrasion tester; density was determined by a hydrometer, following the ISO 4649:1985 standard [22], with three samples tested in each group and the average value was taken; thermogravimetric analysis (TG) was used to assess the thermal stability of the vulcanized rubber using a thermogravimetric analyzer, during which the sample was placed in an alumina crucible, and nitrogen was used as the reaction atmosphere at a flow rate of 50 mL/min. Before the experiment, the system was purged with nitrogen for 10 min to eliminate interference from residual gases, and the heating rate was set at 30 °C/min, with a temperature scanning range from 40 °C to 700 °C.

#### 2.5.2. Characterization of Structure and Morphology

The microstructure of the CBp powder was observed after gold-spraying by scanning electron microscopy (SEM). Fourier transform infrared spectroscopy (FTIR) was used to observe the functional groups of CBp powder; the CBp powder was mixed with potassium bromide to prepare thin slices; then, these were placed on a FTIR spectrometer. Carbon black dispersibility was evaluated by a carbon black dispersity meter, with a dispersion threshold of 23 μm and an exposure time of 40 ms, according to ISO 11345 [23] and ASTM D7723 [24].

## 3. Results and Discussion

### 3.1. SEM Images

**Figure 2:**
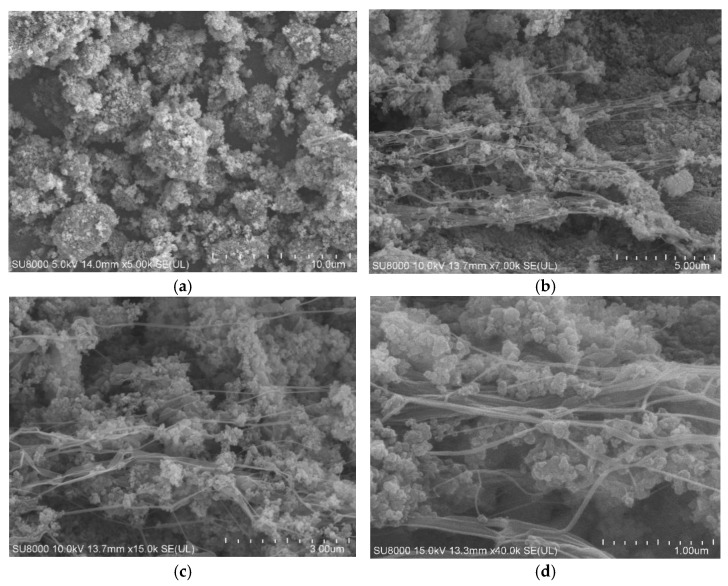
SEM image; (**a**) untreated CBp; (**b**) PTFE1-CBp; (**c**) PTFE3-CBp; (**d**) PTFE5-CBp.

Figure 2 shows the SEM images of untreated CBp and CBp pre-treated with PTFE fibers. From Figure 2a, it can be observed that the dispersion of CBp is relatively uniform, but the connections between the particles are loose, resulting in black gaps. Figure 2b–d displays the CBp after PTFE treatment, where fibrous links between the CBp molecules can be clearly seen, forming a network structure. This structure allows for a tighter and more secure binding of the CBp particles. As PTFE is a high-molecular-weight polymer [25], its chain structure is stable. Under the shear action of the high-speed grinder blades, the internal molecular chains experience changes in stress; some chains are elongated and aligned, while others may break, gradually forming a fibrous structure. These fibers, exhibiting higher tensile strength, can slide past one another when stressed, thereby dispersing stress concentration points. This structure not only strengthens the binding of the CBp particles to form a denser network but also preserves the original chemical properties of PTFE, enhancing the mechanical properties of vulcanized rubber.

### 3.2. Fourier Transform Infrared Spectroscopy

**Figure 3:**
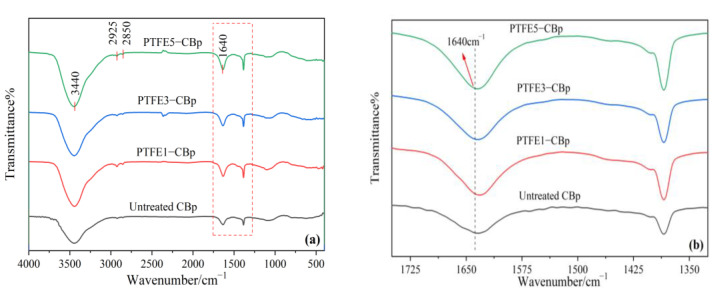
(**a**) FTIR profiles of the four types of CBp in the range of 400−4000 cm^−1^. (**b**) Localized magnification of 1350−1750 cm^−1^.

Figure 3 shows the FTIR profiles of CBp after four different pre-treatment methods. The characteristic peaks at 1640 cm_−1_ and 3440 cm_−1_ correspond to hydroxyl groups, while the peaks at 2850 cm_−1_ and 2925 cm_−1_ are associated with the symmetric and asymmetric stretching vibrations of methyl groups (-CH_3_) [26,27,28]. From the analysis of Figure 2a,b, the characteristic peaks for hydroxyl groups at 1640 cm_−1_ and 3440 cm_−1_ gradually strengthen. This enhancement is due to the pre-treatment processes of PTFE1-CBp, PTFE3-CBp, and PTFE5-CBp in the high-speed grinder, where the rapid rotation of the blades leads to frequent collisions and friction between the CBp particles. This also causes the PTFE to undergo fiber formation, which increases the contact area between the fibrous PTFE and CBp. Within the enclosed chamber, as the temperature gradually rises and the pressure increases, this internal environment enhances the reactivity of the CBp molecules, reduces their inert structure, and promotes oxidative reactions, resulting in the generation of more hydroxyl functional groups.

### 3.3. Filler Dispersion

**Figure 4:**
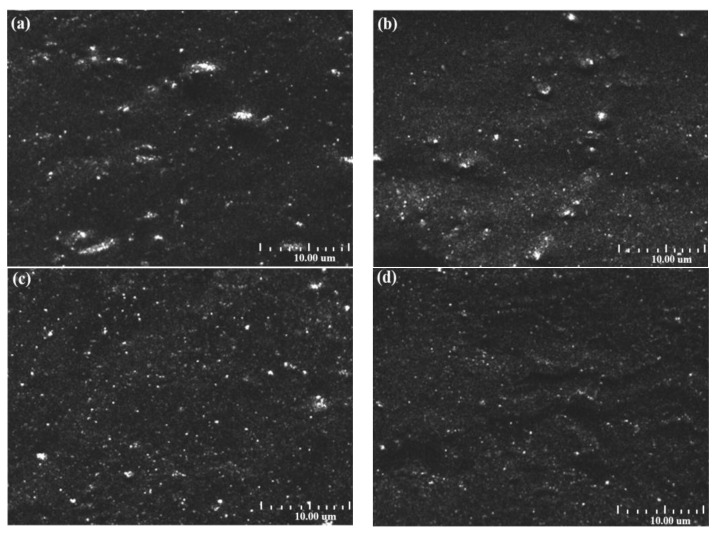
Dispersion image; (**a**) untreated CBp/NR composites; (**b**) PTFE1-CBp/NR composites; (**c**) PTFE3-CBp/NR composites; (**d**) PTFE5-CBp/NR composites.

The carbon black dispersion instrument can analyze particle size, morphology, and distribution to determine the dispersion level of carbon black aggregates [29]. Figure 4 shows the dispersion grades of CBp treated with different amounts of PTFE fibers in an NR matrix, with the white spots representing reflected light from the carbon black agglomerates, and all the data in Table 4 are automatically obtained from the carbon black dispersibility tester. Combining Figure 4 and Table 4, it is evident that in the untreated CBp/NR composites, the dispersion of carbon black in the rubber matrix is poor, with several large white agglomerates present. The average particle size of the agglomerates is 3.4 μm, and the white area accounts for 13.6%. In the PTFE1-CBp/NR composites, the average particle size of the agglomerates is 3.5 μm, with the white area accounting for 14.3%. In the PTFE3-CBp/NR composites, the average particle size is 3.2 μm, and the white area accounts for 12.2%. In the PTFE5-CBp/NR composites, the dispersion level reaches 5.2, with the white area accounting for only 9.6%. Compared to the untreated CBp/NR composites, the dispersion level of PTFE5-CBp/NR improved by 4%, and the white area decreased by 30%. The dispersion images show no significant large white agglomerates, indicating that the filler is evenly dispersed without obvious aggregation. This shows that the addition of PTFE effectively reduces the agglomeration of fillers. The PTFE fibers help encapsulate carbon black during the cross-linking process, reducing the aggregation of carbon black molecules and improving their dispersion in the rubber matrix, ensuring a good mix of all components.

### 3.4. Payne Effect

**Figure 5:**
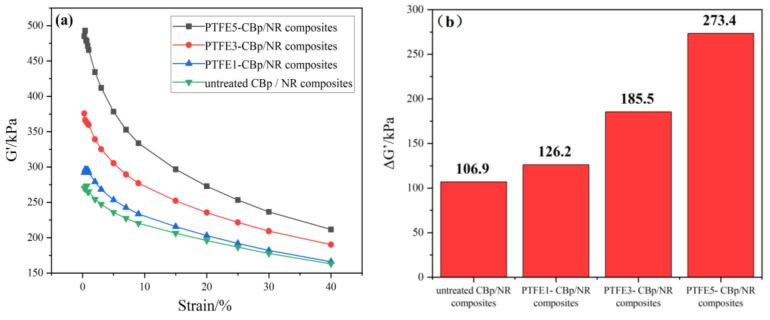
(**a**) Variation in the storage modulus versus strain. (**b**) The change in storage modulus.

Figure 5 shows the curves of the storage modulus (G’) as a function of strain for the vulcanized rubber treated with different amounts of PTFE fibers. G’ represents the storage modulus of the vulcanized rubber, reflecting its ability to storage energy under shear. The ΔG’ represents the change in the storage modulus; a larger ΔG’ indicates a more pronounced Payne effect [30,31]. The Payne effect reflects the interactions between fillers and rubber, and it is linked to the disruption and reorganization of the filler network, interactions between rubber chains and fillers, and the entanglements of rubber chains [31,32]. From Figure 5b, the untreated CBp/NR composites show the lowest ΔG’ at 106.99 kPa, while the ΔG’ values for PTFE1-CBp/NR, PTFE3-CBp/NR, and PTFE5-CBp/NR composites significantly increase, showing increases of 18%, 73%, and 156% compared to the untreated CBp/NR composites, respectively. The results show that with PTFE increases, the Payne effect of PTFE-CBp/NR composites increases. This phenomenon is attributed to PTFE ingas a filler, enhancing the filler–filler network within the rubber matrix [33], thereby leading to an increased in Payne effect.

### 3.5. Mooney Viscosity and Vulcanization Characteristics

Table 5 shows the Mooney viscosity and curing characteristics of the compounds prepared with different amounts of PTFE treatment. Mooney viscosity reflects the flowability of rubber compounds; higher Mooney viscosity corresponds to lower flowability [34]. Compared to the untreated CBp/NR composites, the Mooney viscosities of PTFE1-CBp/NR, PTFE3-CBp/NR, and PTFE5-CBp/NR composites increased by 14%, 53%, and 62%, respectively. As the amount of PTFE increases, the fibers disperse evenly in the rubber matrix and form a skeletal structure, leading to an increase in the strength of the vulcanized rubber and, consequently, increased Mooney viscosity.

M_L_ represents the minimum torque; a higher M_L_ value indicates lower flowability of the compound [35]. From Table 5, it can be seen that as the amount of PTFE increases, the M_L_ value gradually rises, reflecting a decrease in the compound’s flowability and processability. This is due to the fibrous PTFE forming a network structure that tightens the dispersed CBp molecules, causing the polymer chains in the rubber to bond more closely together. Additionally, the high molecular weight of PTFE, approximately 4.5 × 10⁷ [36], leads to longer molecular chains, which limits their movement and further reduces flowability.

M_H_ represents the maximum torque, and the difference (M_H_ − M_L_) is related to the crosslink density of the rubber [35]. A larger M_H_ − M_L_ value indicates a higher crosslink density. From the data in Table 5, it is evident that as the amount of PTFE increases, the values of M_H_ and M_H_ − M_L_ first increase and then decrease, reaching a peak in the PTFE3-CBp/NR composites. This indicates that the vulcanized rubber with 3 phr of PTFE has the highest crosslink density and curing degree. When the amount of PTFE reaches or exceeds 3 phr, it can generate localized stress points within the rubber, disrupting the continuity of the rubber matrix and leading to a reduction in crosslink density.

t_10_ represents the initial vulcanizing time, which is related to the scorch time of the compound. t_90_ refers to the duration needed for the vulcanized rubber to achieve its optimal performance [35]. From Table 5, the t_90_ values for the PTFE-modified vulcanizates were found to decrease, which is attributed to the high strength of PTFE. When added to the rubber, PTFE enhances the compound, thereby shortening the crosslinking time. Moreover, the uniform dispersion of PTFE in the rubber matrix reduces the agglomeration effect of CBp, resulting in more efficient and uniform heat transfer during the curing process, thus improving curing efficiency.

### 3.6. Mechanical Properties

**Figure 6:**
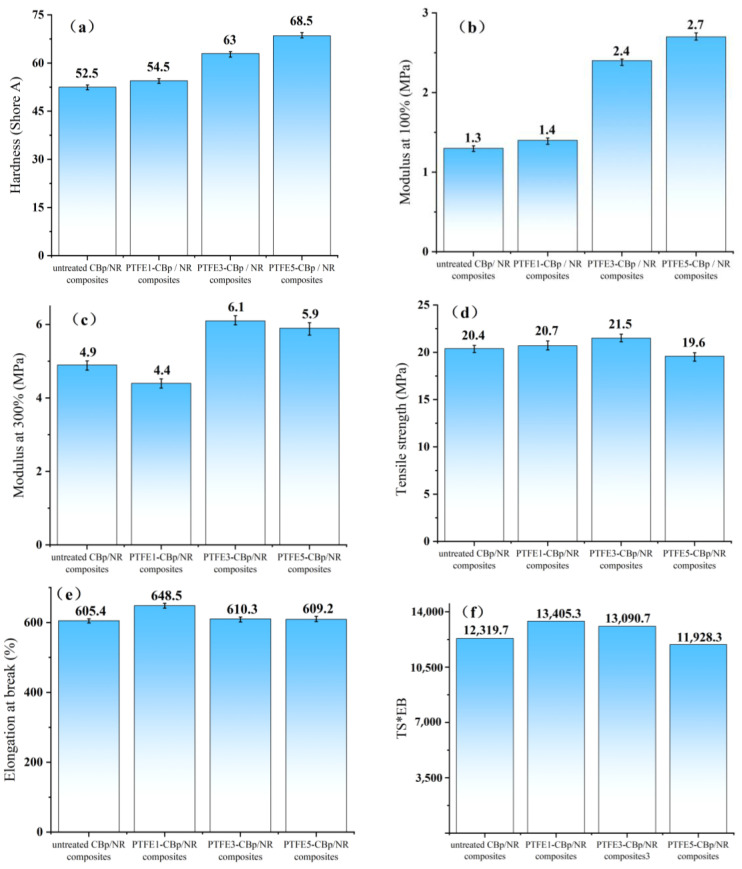
Mechanical properties of four NR composites: (**a**) Hardness; (**b**) Modulus at 100%; (**c**) Modulus at 300%; (**d**) Tensile strength; (**e**) Elongation at break. (**f**) TS*EB.

Figure 6 presents the mechanical properties of vulcanized rubbers prepared with different amounts of PTFE treatment. In Figure 6a, it is shown that as the amount of pre-treated PTFE increases (1 phr, 3 phr, and 5 phr), the hardness of the vulcanized rubber significantly improves compared to the untreated CBp/NR composites. Figure 6b also reflects the increase in modulus at 100%. Compared with untreated CBp/NR, the modulus at 100% for PTFE3-CBp/NR and PTFE5-CBp/NR increased by 89% and 113%, respectively. From Figure 6c,d, it is evident that the PTFE3-CBp/NR composites exhibit the best performance in terms of modulus at 300% and tensile strength, increasing by 24% and 5%, respectively, compared to the untreated CBp/NR composites. Figure 6e,f shows the elongation at break and toughness coefficient (the product of tensile strength and elongation at break), respectively. The highest values for both the elongation at break and toughness coefficient were observed for 1 phr, with increases of 7% and 9% compared to untreated CBp/NR composites. During the high-speed shear modification of PTFE-CBp, the CBp is affected by shear forces, which raise the temperature inside the chamber and lead to the detachment of ash from the surface of CBp, reducing its inertness and exposing more active sites. Meanwhile, the PTFE fibers formed under high shear can uniformly disperse between the CBp molecules, creating a uniform and dense skeletal structure. The PTFE fibers act as a bridge between CBp and NR, enhancing the mechanical properties. However, in the PTFE5-CBp/NR composites, both modulus at 300% and tensile strength show a decline. This is primarily due to the excessive amount of PTFE when the quantity is ≥3 phr, which may weaken the continuity of the rubber matrix and lead to some agglomeration, resulting in stress concentration. When subjected to external forces, these stress concentration points are more likely to cause local rupture or deformation of the material. Additionally, non-fibrous PTFE particles hinder the bonding between the fillers and the rubber matrix, which also affects the reinforcing effect of the rubber.

### 3.7. Density and Wear Resistance

**Figure 7:**
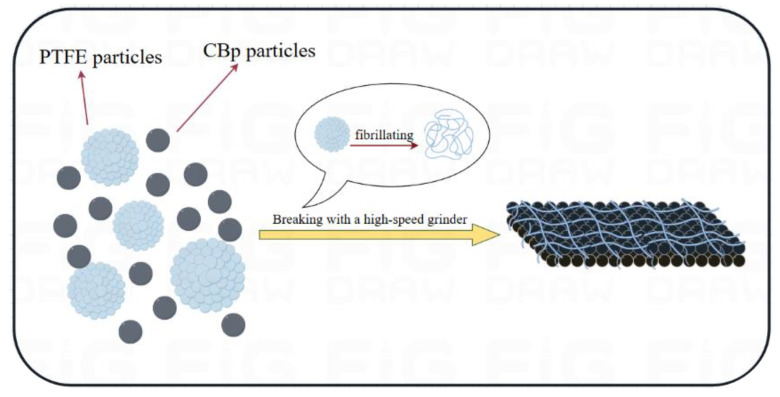
Physical changes in PTFE-CBp during the process.

**Figure 8:**
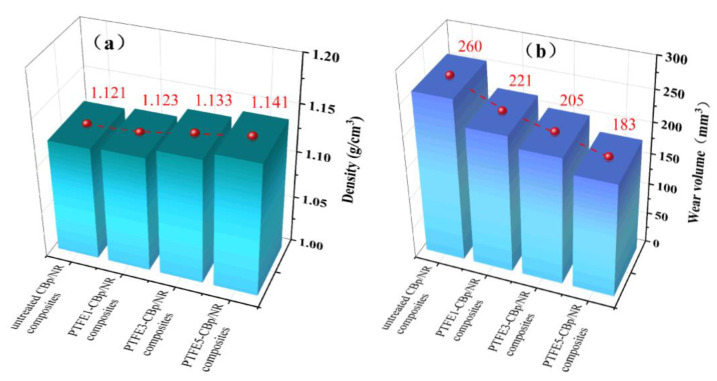
(**a**) Density of four NR composites; (**b**) wear amount of four NR composites.

Figure 7 shows the physical changes of PTFE-CBp during the high-speed crushing process. Figure 8a shows the density of four different vulcanized rubbers. It can be observed that as the PTFE content increases, the density of the NR composites gradually rises. This is attributed to the higher density of PTFE (2.2 g/cm³) [37,38]. Additionally, the fibrous network structure of PTFE enhances the tightness of connections between CBp, reducing the porosity of the NR composites and consequently increasing their density. Figure 8b shows the wear amounts of four different vulcanized rubbers. Analyzing the data reveals that the untreated CBp/NR composites exhibit the highest wear amount and the poorest wear resistance. As the amount of PTFE increases, the wear amount of the vulcanized rubber gradually decreases, indicating an improvement in wear resistance. Compared to the untreated CBp/NR composites, the wear amounts of PTFE1-CBp/NR, PTFE3-CBp/NR, and PTFE5-CBp/NR composites decreased by 15%, 21%, and 30%, respectively. PTFE inherently possesses self-lubricating properties and a low coefficient of friction, and its chemical characteristics remain unchanged after fiberization [39,40]. When the pre-treated PTFE fibers and CBp are mixed with NR during the compounding process, the self-lubricating characteristics of PTFE are retained, reducing wear consumption. Additionally, the PTFE fibers form a network structure within the rubber, which can absorb some of the stress under external forces, thereby minimizing damage to the rubber structure and enhancing the wear resistance of the compounded rubber.

### 3.8. Thermal Stability

**Figure 9:**
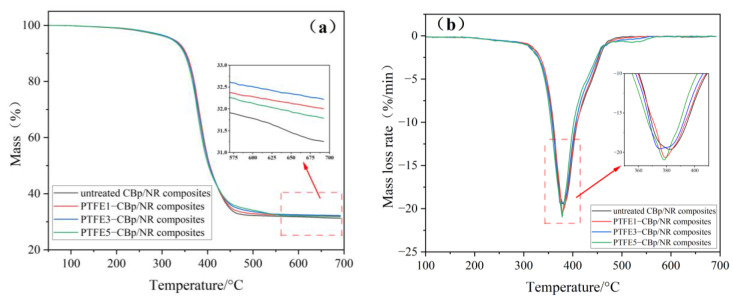
(**a**) TG curve of the vulcanizates. (**b**) DTG curve of the vulcanizates.

Figure 9 shows the TG-DTG curves of the vulcanized rubber, with a testing temperature range from 40 °C to 700 °C. The vulcanized rubber samples gradually decompose and vaporize as the temperature rises, and the mass at around 700 °C represents the ash content [41]. From Figure 9a, it can be seen that for the four different vulcanized rubber samples, the mass loss remains between 5% and 10% when heated to 350 °C, indicating that these vulcanized rubbers exhibit good thermal stability within this temperature range. The mass loss at this stage is primarily due to the removal of physically adsorbed water. In the temperature range of 350 °C to 600 °C, the mass loss of all four samples increases significantly, and the DTG curve shows a distinct endothermic peak, with a mass loss of approximately 65%. This stage of mass loss is attributed to the thermal degradation process, where the chemical bonds within the polymer material break, mainly due to the removal of chemically adsorbed water and dehydroxylation of fillers. From the enlarged image in Figure 9b, it can be observed that around 380 °C, the decomposition rate of the PTFE5-CBp/NR composite material reaches its maximum, with a significant increase in the peak value of the DTG curve, indicating that the thermal degradation rate of this composite is most intense near this temperature. Finally, when the temperature rises to 600 °C to 700 °C, the residual mass of the four samples stabilizes, with the remaining solid material consisting mainly of ash.

Analyzing Table 6 reveals that the untreated CBp/NR composite exhibits the lowest initial decomposition temperature, which is 365.9 °C. In contrast, the PTFE1-CBp/NR composites show an increase in their initial decomposition temperatures by 5.7 °C when compared to the untreated CBp/NR composite. This indicates that the addition of PTFE fibers enhances the initial decomposition temperature of the vulcanized rubber. The reason lies in the molecular structure of PTFE, where the main chain consists of carbon–carbon (C-C) bonds, with each carbon atom bonded to two fluorine atoms (C-F); the bond strength of C-F can reach 485 kJ/mol [42]. After the pre-treated PTFE-CBp is fully combined with NR, the initial decomposition temperature of the compounded rubber increases, thereby improving the thermal resistance of the rubber material and delaying the high-temperature aging process. The trend in the residual mass at 700 °C is consistent with the changes in the initial decomposition temperature. After adding PTFE fibers, the residual mass at 700 °C also increases. This is attributed to the high melting point of PTFE, which is 327 °C [43]. The dense protective layer formed by the fluorine atoms provides a barrier around the carbon chains, resulting in the retention of more solid residues under high-temperature conditions at 700 °C, further enhancing the thermal stability of the rubber products.

## 4. Conclusions

This study utilizes the high-energy shear force of a high-speed grinder to modify and characterize PTFE and CBp, promoting the alignment of carbon black particles in specific directions to form fiber-like structures. This research investigates the filler dispersion degree, Payne effect, vulcanization characteristics, mechanical properties, and wear resistance of NR composites filled with PTFE-CBp. FTIR analysis indicates that high-energy crushing enhances the surface activity of CBp. Compared to untreated CBp/NR composites, the hardness of PTFE1-CBp/NR, PTFE3-CBp/NR, and PTFE5-CBp/NR composites increased by 4%, 20%, and 30%, respectively; the modulus at 100% increased by 3%, 89%, and 113%; and the elongation at break also increased. Additionally, the initial decomposition temperature of PTFE1-CBp/NR composites increased by 5.7 °C compared to untreated CBp/NR composites. As the amount of PTFE increased, the modulus at 300% and tensile strength exhibited a trend of first increasing and then decreasing, with the PTFE3-CBp/NR composite showing the best performance. Furthermore, the wear resistance improved by 21%, effectively reducing the wear of the rubber matrix. This provides a theoretical basis for the preparation of low-wear rubber composites using CBp.

## Figures and Tables

**Table 1:** Experiment formulation of rubber composites.

Material	Amount (phr ^1^)	Material	Amount (phr)
NR	100	Sulfur	2.5
CBp	50	DM	0.6
ZnO	5	SAD	3
PTFE	0/1/3/5		

^1^ phr: the number of additives per 100 parts of rubber.

**Table 2:** The used apparatus and corresponding manufacturers.

Equipment	Model	Manufacturer
High-speed grinder	WPB12J30	Meiyu, Fuzhou, Fujian, China
Fourier transform infrared spectrometer	IS50	Thermo-Scientific, Waltham, MA, USA
Rotorless rheometer	MDR-C	Alpha Technologies, Wilmington, DE, USA
Mooney viscometer	PREMIER MV	Alpha Technologies, Wilmington, DE, USA
Rubber process analyzer	RPA-2000	Alpha Technologies, Wilmington, DE, USA
Universal testing machine	Instron-3365	U-CAN, Norwood, MA, USA
Carbon black dispersibility tester	Disper GRADER	Alpha Technologies, Wilmington, DE, USA
Shore A hardness tester	H17A	Wallace instruments, Dorking, Surrey, UK
Thermogravimetric analyzer	209F3	NETZSCH, Selb, Bavaria, Germany
Rotary roller abrasion tester	SS-5643-D	Songshu, Dongguan, Guangzhou, China

**Table 3:** Materials’ naming and pre-treatment process.

	Untreated CBp	PTFE1-CBp	PTFE3-CBp	PTFE5-CBp
PTFE(phr ^1^)	0	1	3	5
CBp(phr ^1^)	50	50	50	50
Pre-treatment process		Add PTFE and CBp simultaneously into the high-speed grinder, with a rotational speed of 25,000 rpm and a processing time of 20 min.		

^1^ phr: the number of additives per 100 parts of rubber.

**Table 4:** The dispersion data of the studied composites.

	Untreated CBp/NR Composites	PTFE1-CBp/NR Composites	PTFE3-CBp/NR Composites	PTFE5-CBp/NR Composites
Dispersion level	5.0	4.5	5.5	5.2
Average particle size of agglomerates/um	3.4	3.5	3.2	3.2
White area/%	13.6	14.3	12.2	9.6

**Table 5:** Vulcanization characteristics.

Test Project	Untreated CBp/NR Composites	PTFE1-CBp/NR Composites	PTFE3-CBp/NR Composites	PTFE5-CBp/NR Composites
M_L1+4_ (100 °C)	36.0	40.9	55.1	58.3
M_L_/(dN·m)	1.1	1.3	1.7	2.0
M_H_/(dN·m)	10.6	10.7	11.4	11.3
M_H_ − M_L_/(dN·m)	9.5	9.4	9.7	9.3
t_10_/(min)	1.8	1.7	1.7	1.6
t_90_/(min)	11.5	11.4	9.9	10.7

**Table 6:** TG date of the vulcanizates.

	Untreated CBp-CBp/NR Composites	PTFE1-CBp/NR Composites	PTFE3-CBp/NR Composites	PTFE5-CBp/NR Composites
Onset decomposition temperature/(°C)	365.9	371.6	366.6	371.2
Residual mass at 700 °C/(%)	31.3	32.0	32.2	31.8

## Data Availability

The original contributions presented in this study are included in the article. Further inquiries can be directed to the corresponding author.
